# A Postscript to the Report on Appendicitis*See Johns Hopkins Hospital Bulletin, June-July, 1894.

**Published:** 1895-01

**Authors:** W. S. Halsted

**Affiliations:** Baltimore, Md.; Surgeon-in-Chief, etc., Johns Hopkins Hospital


### A POSTSCRIPT TO THE REPORT ON APPENDICITIS.*

BY W. S. HALSTED, M. D., BALTIMORE, MD.,

Surgeon-in-Chief, etc., Johns Hopkins Hospital.

Dr. Finney's remarks on the treatment of the wound in cases of appendicitis have been abbreviated so much as possibly to mislead those who are not familiar with our methods. When he speaks of "leaving the abdominal wound open" he means that the wound is drained with gauze, and not that no attempt is made to close it. The fact is that the wound is sewed up tight about the gauze, so tight that it is sometimes necessary to cut one stitch in order to remove the packing. Whenever pus is encountered, either within the appendix or outside of it, the wound is drained. Sometimes one or two narrow strips of gauze are sufficient, sometimes very many broad strips are required. Ordinarily all of the gauze is brought out at one point and between stitches which, as I have said, embrace it snugly. The gauze is used not only for drainage, but quite as much to stimulate adhesions between the coils of intestine which surround it and thus effectually shut off the general peritoneal cavity from its infected portion. The gauze is gently packed about the stump of the appendix, and should reach into every recess of the pus cavity. When the abscess is a large and ramifying one, or when there are several abscesses, we may bring the gauze packing out of the abdomen at more than one point in the wound.

These wounds are closed with mattress sutures; but the sutures are not always buried as they are in all uninfected abdominal wounds which are combletely closed and in which the danger of stitch infection is not so great. The stitches, where they are not buried, are prevented from cutting into the skin by pieces of rubber tubing or of gauze. These wounds should be stitched with great care. All of the divided tissues (the peritoneum excepted) should be included in each stitch unless the stitches are buried. Inasmuch as the muscles retract unevenly, the sewing is sometimes a difficult task. If the wound is sewed in this way, and if sufficient care is exercised to avoid the in-

*See Johns Hopkins Hospital Bulletin, June-July, 1894.

fection of the stitches as they are being introduced and tied, there is little if any danger that a hernia will ensue.*

Even the point at which the gauze traverses the abdominal wall is not a weak one. A connective tissue membrane, the wall of the obliterated sinus, extends from the stump of the appendix to this point in the wound and binds the intestines to each other, and to the under side of the lips of the open part of the wound. The thickness of this membrane depends principally upon the length of time that the gauze is allowed to remain undisturbed. I have found it so strong after ten days that I could with difficulty thrust my finger through it. This membrane atrophies in time. After two years I have found the walls of a sinus to the gall bladder attenuated to little more than a trace.

With our present resources it is not justifiable to attempt to disinfect an abscess cavity of the peritoneum, no matter how infinitesimally small this abscess may be. Bull and two others, whose names I am not at liberty to mention, are probably not the only ones who have furnished disastrous instances of such attempts.

In operations for appendicitis we have always the strangulated stump of the appendix and usually tissues more or less necrotic in its immediate vicinity as a complication. My experiments† demonstrated conclusively the result of inoculation of strangulated tissues in the peritoneal cavity.

The problem is a very different one when we have an abscess in the cancellous tissue of bone or in highly vascular soft parts to deal with. We may safely close such abscess cavities. If, for example, the so-called pyogenic wall of an abscess in muscle is excised and the parts are then thoroughly washed with an antiseptic solution, we may so far inhibit the pyogenic organisms that the tissues or, if there is a dead space, the prolific granulations, assisted possibly by the blood, may altogether destroy them. In the cancellous tissue of bone a cavity large

*At the meeting of the Johns Hopkins Medical Society, November 5, 1894, I presented a case of appendicitis to illustrate our treatment of the incision. Buried sutures of silver wire had been used to bring together the cut edges of the abdominal muscles, and an uninterrupted buried suture of silver wire closed the wound in the skin. The latter suture had already been withdrawn and a fine pink line indicated where the skin incision had been made. A little below the centre of the wound was the orifice of a sinus from which a nar row strip of gauze had just been removed. The cicatrix was three weeks old.

†The Johns Hopkins Hospital Reports. Report in Surgery, I.

enough to hold a hickory nut becomes completely filled with granulations in about three days. Blood clots occupying such cavities, if inoculated with virulent cultures of staphylococcus auıeus, rarely break down. As a rule, the so-called organization of the clot takes place in from two to four days without suppuration. But an abscess in the peritoneal cavity is a very different affair because (1) the wall of the abscess consists in part of strangulated or more or less necrotic tissue which we cannot excise; (2) attempts to disinfect such an abscess would probably be futile and might be worse than futile; (3) failure to disinfect might mean general peritonitis and the death of the patient, and not merely the retardation of healing.

There cannot be a definite incision for appendicitis. In general, if there is a large abscess, the incision should be made as near as possible to the crest of the ileum, so as to diminish the chances of entering the clean peritoneal cavity and to lessen the possibility of a hernia. The muscles are thick in this region, and when divided offer broad surfaccs for coaptation by suture; and if the incision is too close to the ileum to admit of suture, there is little danger of hernia resulting, as we know from a long experience with psoas abscesses, which we open by preference in this region. But the position of the abscess or, if there is no pus or too little pus to be detected, the position of the appendix in the given case should determine the site of the incision. If there is an abscess, the tissues over it should be most carefully studied as they are being incised for signs of in filtration with inflammatory products. A little œdema of the deeper muscles (transversalis or internal oblique) may guide us to a circumscribed spot of adhesion of cæcum or omentum to parietal peritoneum and enable us to empty a large abscess without entering the uninfected part of the peritoneal cavity, or to thoroughly protect the intestines about the encapsulated pus cavity from the danger of infection before the pus is liberated. We place several yards of gauze between the healthy intestines and the abscess before opening the latter.

From a bacteriological point of view, we must often, if not always, inoculate the healthy peritoneum, but thus far we have not in a single instance had peritonitis supervene upon an operation for appendicitis, nor have we a single death to attribute to the operation. In the case of a large abscess, which we have evacuated without entering the uninfected peritoneal cavity, we still hesitate to search for and remove the appendix if its removal would necessitate our entering the clean peritoneal cavity.

When there is little or no pus to be discovered, we make our incision directly over the appendix, which can usually be palpated. Here, too, we try to cut through thick muscles if possible. The instant that the peritoneum is opened, and before it is widely incised, we introduce large towels of gauze, and with these press the intestines over the appendix out of the way and towards the left. When the appendix is nicely exposed and a clear field for operation obtained, we introduce more gauze to serve as an inner lining to the outer ring of gauze. The adhesions which bind down the appendix are then slowly broken up by gentle finger pressure, and if pus is present, it is caught as it leaks out by additional gauze sponges. If the inner layer of gauze packing should by accident become soiled, it is immediately replaced by fresh packing, the opening into the abscess being meanwhile stopped with a gauze sponge. And so, little by little, the abscess is emptied, and finally the appendix removed. After ligating the appendix and its mesentery, we may excise the mucosa which is cut off by the ligature. We never sew up the end of the stump in the infected cases, as some surgeons have advised. This would be a foolish waste of time; for the circulation of the part stitched has been cut off by the ligature applied to the appendix. The gauze for packin gis rubbed full of a mixture of iodoform and bismuth and then sterilized.

